# Microchip Health Monitoring System Using the FLL Circuit

**DOI:** 10.3390/s21072285

**Published:** 2021-03-24

**Authors:** Emmanuel Bender, Joseph B. Bernstein

**Affiliations:** Department of Electrical and Electronic Engineering, Ariel University, Ariel 40700, Israel; josephbe@ariel.ac.il

**Keywords:** FPGA, failure mechanisms, BTI, reliability prediction, prognostics

## Abstract

Here a solution for a Microchip Health Monitoring (MHM) system using MTOL (Multi-Temperature Operational Life) reliability testing assessment data is proposed. The module monitors frequency degradation over time compared to lab tested data. Since trends in performance degradation in recently developed devices have transitioned from multiple failure mechanisms to a single dominant failure mechanism, development of the monitor is greatly simplified. The monitor uses a novel circuit customized to deliver optimum accuracy by combining the concepts of ring oscillator (RO) and phase locked loop (PLL) circuits. The modified circuit proposed is a new form of the frequency locked loop (FLL) circuit. We demonstrate that the collection of frequency degradation data from the ring circuits of each test produces Weibull distributions with steep slopes. This implies that the monitor can predict accurate end-of-life (EOL) predictions at early stages of chip degradations. The design of the microchip health monitoring system projected in this work can have great benefit in all systems using FPGA and ASIC devices.

## 1. Introduction

The semiconductor industry is rapidly generating solutions for problems in virtually every sphere of life. Therefore, accommodable surroundings are only possible when infrastructures include foolproof microchip systems. Chip failure, always damaging, can be catastrophic in critical systems. Consequently, the implementation of dependable prognostic monitors of chip health is essential. Our solution is a chip health monitor based on reliability data trends. Our monitoring senses degradation, by incorporating advanced measuring concepts, while the devices operate under normal conditions.

**Figure 1:**
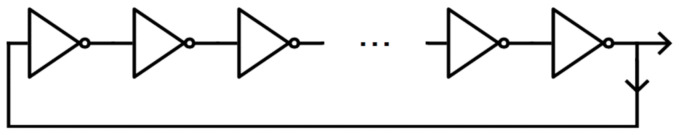
Diagram of a ring oscillator created by closing a chain of inverters.

The health monitor proposed compares on-chip data to lab tested data using the MTOL testing method. MTOL has already been implemented on FPGA boards of different transistor technologies: 45, 28, and 16 nm [1,2]. The MTOL method produces reliability predictions over a wide temperature range (−50 °C to 150 °C) from frequency degradation data of several different stress modes. Data is received by aging ROs, displayed in Figure 1, with multiple voltage, current, and thermal stress modes. Current stress is generated from device transitioning. A set of multi-sized concurrently oscillating rings are designed in the FPGA device to produce a large range of frequencies. Therefore, data results that show frequency dependency bare witness of a failure mechanism (FM) that is stimulated by current. Hot carrier injection (HCI), current triggered, appears with the increase in frequency [3]. HCI, a FM with a negative activation energy (E_A_), is accelerated by sub-zero temperatures [4]. Bias temperature instability (BTI) is not frequency dependent, since it is only voltage related. It has a positive E_A_, since it is present at high temperatures and voltage [5,6]. Electromigration (EM), also current dependent, is accented by high temperatures and high frequency [7,8]. From the data, proportionate weights of the FM are solved in a matrix of extrapolated time to failure (TTF) values to create the reliability portrait [9,10]. The results of these studies present a clear transition from the earlier technologies represented by 45 nm, having multiple dominant FM: BTI, EM, and HCI, to 28 nm and 16 nm showing the presence of a single dominant FM, which is BTI.

**Figure 2:**
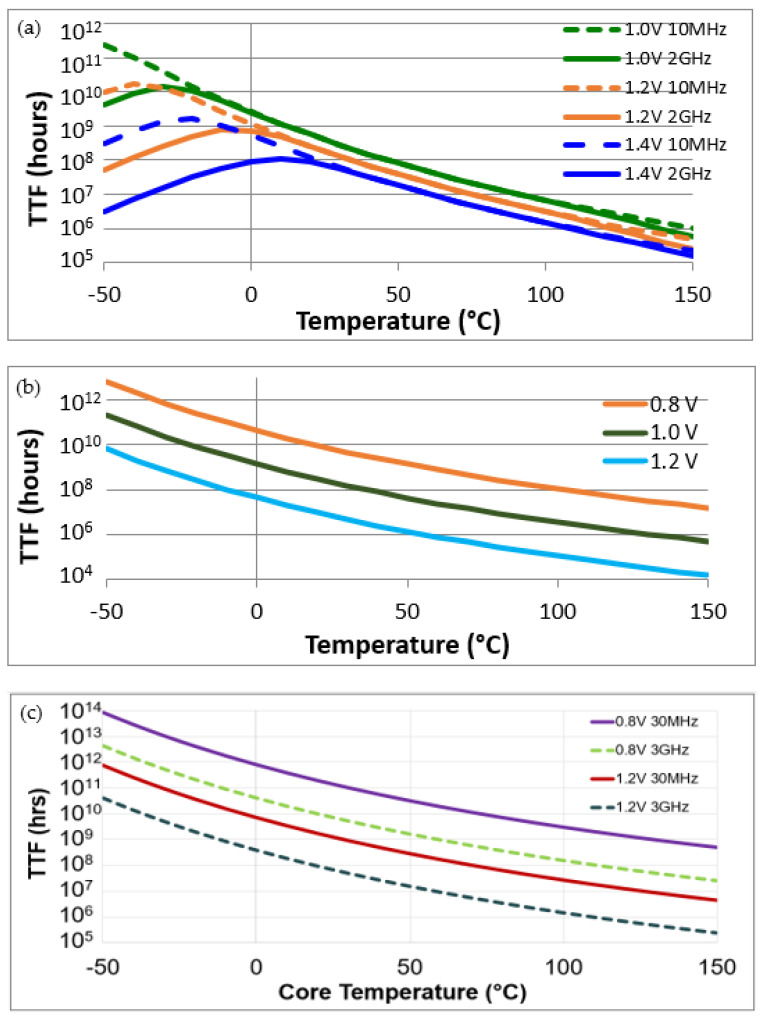
A display of a set of reliability profile curves for 45 nm (**a**), 28 nm (**b**), and 16 nm (**c**) technologies.

The full reliability profile is displayed in Figure 2 for 3 different technologies of FPGAs: 45 nm, 28 nm, and 16 nm. This summary shows the evolution of chip design as the dimensions of the cells are reduced. In Figure 2a, the 45 nm profile attests to multiple mechanisms: EM, BTI, and HCI. The dip in the curve in low temperatures, which is accented in high frequencies, is the HCI signature. There is a forking in high temperatures. BTI is responsible for the performance drop in low frequencies [11,12,13]. EM causes degradation in high frequencies [14]. In the profile for 28 nm (2b), EM and HCI are negligible leaving only BTI. Frequency does not play a strong role (if any) in the decline of the devices. In 16 nm FinFETs (2c), BTI continues to be the dominant mechanism. In contrast to 28 nm, frequency affects the device lifetime due to the self-heating effect (SHE) [1]. As device dimensions continue to undergo reductions, the trends shown in the study are conserved. Other studies show that the impact of SHE increases considerably in 7 nm and 5 nm technologies [15]. In addition, as the substrate is more isolated from the bulk in designs such as nanotubes and GAAFETs, self-heating problems grow [16]. In any case, the consequences of SHE worsen the effects of BTI [17].

Considering the above, chip monitoring in leading-edge technologies can be accomplished using a single-parameter control set, allowing the development of a monitoring solution to be relatively simple. Our studies show the benefits of using RO type circuits to find the failure behavior of the devices tested. Likewise, such orientations are very suitable for a health monitoring system design. By implementing the FLL circuit in place of the RO circuit, the resources required to create a monitor are reduced considerably. The proposed FLL circuit differs from FLL circuits implemented in other studies [18]. Previous FLL implementations are designed to keep the frequency constant by correcting the internal voltage. In the case of health monitoring systems, it is imperative to measure the frequency degradation to sense the health of the microchip. Our FLL circuit innovation, which measures frequency changes over time to monitor the performance of the system, has not been introduced in previous studies. Therefore, the FLL circuit proposed in this work is the seamless solution for anticipating early microchip failures.

In the following section, the procedure for extracting TTF figures from RO test systems is detailed. In the discussion, we demonstrate how the process requires the gathering of data from a large number of circuits. This poses a serious design challenge, as processing of a large amount of frequency data will be very costly to microchip space and power resources. After that, in Section 3, the FLL circuit is proposed as a solution to the problem. Section 4 details the health monitoring system formulated from the concepts discussed previously.

## 2. Weibull Distribution Tapering by Increasing Devices

**Figure 3:**
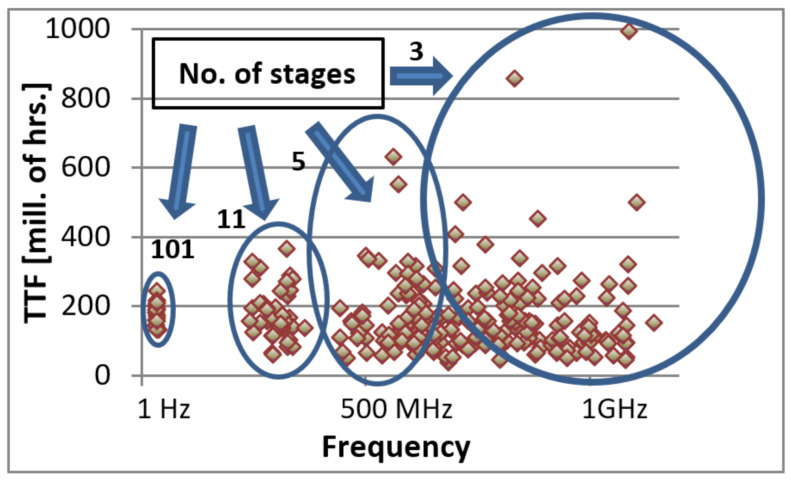
An example of a time to failure (TTF) to frequency plot. The frequency varies with number of stages in the rings (listed in the figure). The TTF values become more dispersed with increase of frequency.

Prior to the FLL circuit design, frequency dependency was checked using variable-length ROs, as mentioned previously. Acquisition of accurate results required large amounts of data. Frequency of each ring circuit is dictated by the number of stages included in the ring. More stages create a longer propagation delay, decreasing the frequency. Figure 3 displays an example of a TTF value to ring frequency plot of the test data. The data fans out in the higher frequency rings. The rings with many stages have a much tighter distribution than those with few stages. Since the TTF values in higher frequency rings are very dispersed, 160 3-stage ring circuits were programmed into the devices to receive a precise average. For 11-stage rings, similar accuracy is received from averaging 20 circuits.

**Figure 4:**
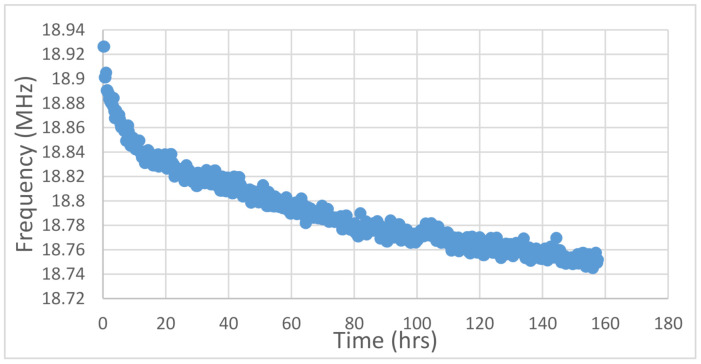
Plot of frequency degradation of a ring oscillator (RO) composed of 101 stages in hours. The data as-is cannot be extrapolated to a time to failure value without being converted to the correct time law.

**Figure 5:**
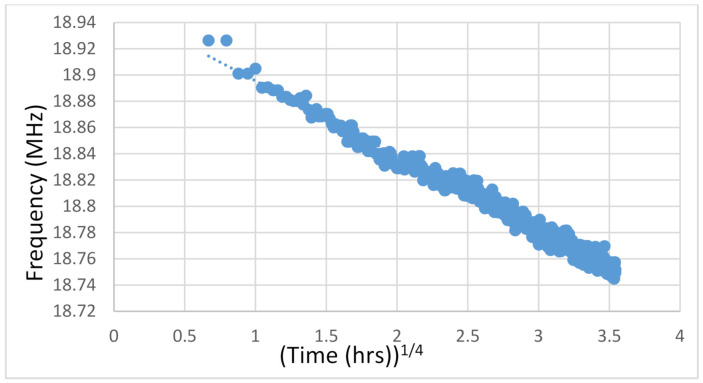
Plot of frequency degradation of a RO composed of 101 stages. It is plotted to a 4th root power law creating a straight line from a curve.

The investigation of the dispersion phenomenon starts with calculating the TTF values of the data. Figure 4 shows the frequency degradation data of a single 101 stage ring circuit stress tested for about 160 h. Extrapolation is realized by transforming the degradation curve into a straight line. The degradation processes of failure mechanisms are not linear. Since most material damages develop due to a diffusion process, the deterioration will advance with time raised to some fraction. For example, BTI arises due to hole-assisted breaking of Si-H bonds at the Si/SiO_2_ interface [19]. Different failure mechanisms have different time scales of degradation. Based on empirical studies, HCI degradation can be transposed to a square root time scale [20] and BTI to a fourth root [21]. From the stress conditions of the test in Figure 4, as well as from the results of performing an RMS fit, the power in the test is determined to be a 4th root law. Figure 5 shows the same results from Figure 4 converted into a fourth root time scale. The result is a uniform slope over all of data. Analysis of the degradation data is actualized by extrapolating the frequency decrease to the point where the device is non-functional. Our definition of chip failure is a 10% depletion in performance. That level deterioration will cause device to be inoperable according to most standards.

**Figure 6:**
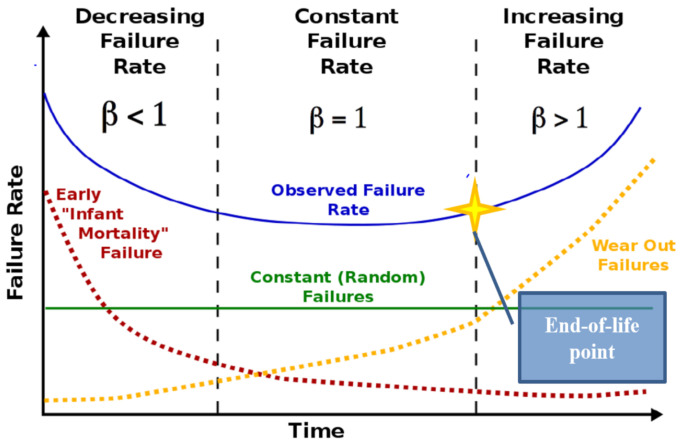
The Bathtub reliability curve is used to describe device failure-rate characteristics for nearly all devices. The relationship of Weibull plot slopes to the bathtub curve is set by the value of β.

Further detail is received by studying the Weibull distribution of each group of ring sizes: 3, 5 and 11 inverters. The Weibull distribution [22], named after the Swedish Professor Waloddi Weibull, is perhaps the most used distribution for lifetime data analysis. While being straightforward compared to other distributions formulas, the Weibull distribution is also versatile enough for analyzing diverse types of aging phenomenon. We found a direct correlation between the number of stages in the rings to the Weibull distribution slope for the *TTF* of that group of rings. The level of randomness of the *TTF* values is indicated by the slope, *β*, from the Weibull Reliability Probability distribution as detailed in Equation (1) [23]:

(1) R(t) =e−(tθ)β

where *θ* is the characteristic failure time. *β* is the slope of the distribution also referred to as the shape parameter. When the *β* slope is about 1, the system exhibits a failure distribution that is almost completely random. Distributions where the *β* slope is higher than 1, illustrate a more deterministic failure characteristic. As the shape parameter increases, the failure distribution approaches a single failure time. This transition is clearly differentiated in the frequently used “Bathtub Curve” displayed in Figure 6 [24].

The accented point on the right side of the curve is the transition from a constant failure rate to the end-of-life time of the device. Our assumption, based on the orientation of the data displayed in Figure 3, is that Weibull distributions categorized by ring size will reveal a correlation between *β* slope and the number of stages in a ring. The procedure for plotting a Weibull distribution is provided in the steps below. Equation (1) is rearranged to isolate *β*. More appropriate names are used for the *R(t)* and *θ* parameters creating the following formula:

(2) $$\ln\left(-\ln\left(1-R\left(t\right)\right)\right)\;=\beta *\ln\left(\theta \right)\to \ln\left(-\ln\left(1-\frac{failure\#}{\#\;of\;rings}\right)\right)\;=\beta *\ln\left(TTF\right).$$

The ring data for each ring size is categorizing by *TTF* value starting from the shortest failure time. Calculation of the TTF value, found on the right side of the equation, is performed in two steps:

1. The slope of the degradation curve is calculated with the following Excel^TM^ formula:

(3) normalized slope (NS)=−slope(ln(ring frequncy), time [4th root]).

2. The formula for calculating degradation down to 90% (*TTF*), assuming the n-root law of 0.25, is:

(4) TTF=(0.1NS) 4.

The Weibull slopes are presented by plotting the “Weibit”, which is based on the number of failures as follows:

(5) $$Weibit=\ln\left(-\ln\left(1-\frac{failure\#}{\#\;of\;rings}\right)\right).$$

**Figure 7:**
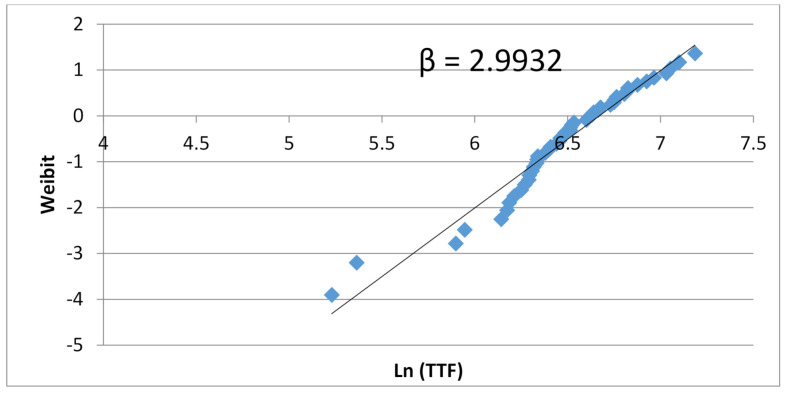
Weibull distribution created using 3 stage ring oscillators on 45 nm technology with stress conditions of 35 °C oven temperature and 1.2 V core voltage.

The x-axis is plotted to: ln(*TTF*) and the y-axis is the Weibit. The slope of the plot is *β* as presented in Figure 7.

**Figure 8:**
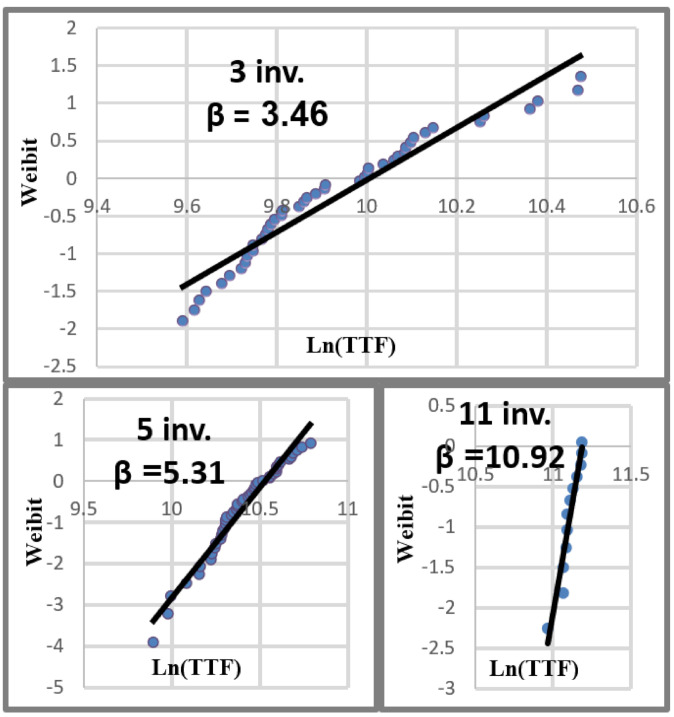
Weibull plots of 3, 5, and 11 inverter ring oscillators. The results demonstrate the direct correlation between number of inverters and Weibull slope β.

A collection of Weibull distributions of 3, 5, and 11 stage rings is displayed in Figure 8 in the following page. A clear one-to-one correlation between the number of stages in the rings and the *β* slope appears. As will be explained later in an analytical study, these results produce a good practical example of the central limit theorem (CLT). Based on Drenick’s deduction, one can expect a completely random failure rate for each stage [25]. In any case, there is a large difference between the TTF distribution of small rings to large rings. The explanation is that, in the small rings the output signal is an average of few stages, resulting in highly diverse TTF values. Larger rings produce a TTF value averaged over more stages, producing a tightly bound distribution of TTF values.

The same conclusion is found by inspecting the ring circuits analytically with reliability models. The Weibull function takes only the extreme value approach. In other words, only TTF values much smaller than the mean time to fail (MTTF) are considered. This allows the use of a constant failure rate model. The reliability function for a single element, *R(t)* and the failure function, *F(t)* are listed in Equation (6). This equation is built from the first order Poisson function. As the conditions of the system are time-independent, the failure rate *λ* is constant. Thus, we have:

(6) $$R\left(t\right)=e^{-\lambda t},\;F\left(t\right)=1-e^{-\lambda t}$$

**Figure 9:**
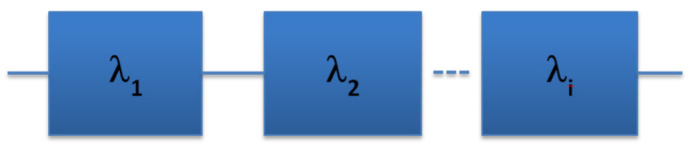
Series or weakest link failure system model.

Equation (6) describes a single element system. Ring oscillators include multiple elements. It is imperative to clarify what behavior best describes how the elements contribute to the failure of the system. The seemingly most obvious fit for a failure system model for microelectronic devices is the series system model [26]. For example, FPGA devices, consisting of a matrix of logical elements called lookup tables (LUTs), will only operate if all the LUTs are functional. Therefore, just like the strength of a chain is as strong as its weakest link, the reliability of a FPGA is only as robust as its worst LUT [27]. The diagram below (Figure 9), gives a graphic representation of the series system model in reliability terms.

The total reliability is described as follows:

(7) $$R\left(t\right)=\left(e^{-\lambda_{1}t}\right)\left(e^{-\lambda_{2}t}\right)\ldots (e^{-\lambda_{i}t})\;\sim \;e^{-i\lambda_{1}t}\;.$$

To suggest that the rate of failure in practice can be developed from this model is contradictory to evidence in the field. One would be forced to say that the TTF values decrease proportionally with the number of transistors or gates in the device. The series model suggests that the failure rate should increase as a function of the number of devices in the system. Since device numbers are increasing exponentially through time, chip failure rates should also be increasing at a comparable rate. Based on in-field data, this is not the case. Therefore, the serial system model alone does not properly reflect the failure characteristics of a full microprocessor.

**Figure 10:**
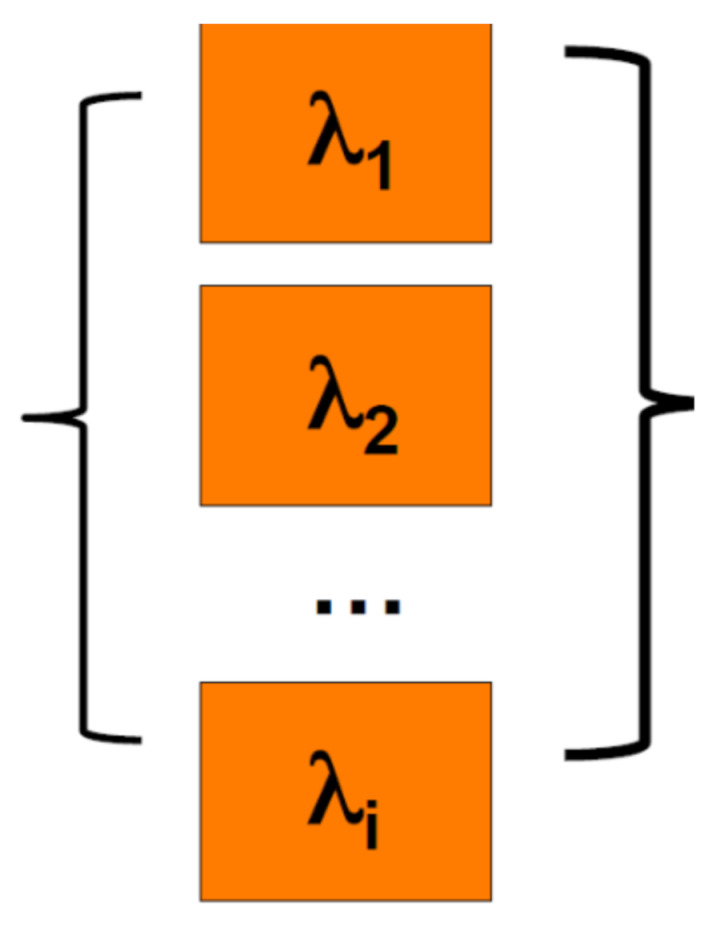
Failure of devices oriented in a parallel system.

The parallel system model is visualized in the in Figure 10.

In a Parallel system the system only fails after all the components have failed. Assuming that the failure probability of each component is random, the probability for a single element is a Poisson process with a failure rate of *λ_i_*. One can ask, what is the justification for describing the microelectronic device failure behavior as a parallel system? It seems obvious that each element in the chip is prone to fail and thus causing the whole device to fail. In other words, it can be defined as a classical series system. This perspective is misleading because it suggests that elements in a device are prone to have catastrophic or complete failures. This is not commonly observed in test data. Rather, performance degrades disproportionately for each different element. Since the different elements in a logical path influence the response time of the logical path, they average together into a comprehensive failure rate *λ* value. Consequently, the interaction between the stages in a ring can best described as a parallel failure system. This is because the stages become averaged together to generate the TTF of the ring. The failure probability of the parallel system is defined as:

(8) $$F\left(t\right)=\left(1-e^{-\lambda_{1}t}\right)\left(1-e^{-\lambda_{2}t}\right)\ldots \left(1-e^{-\lambda_{i}t}\right).$$

In the case where the variance in the rate (*λ_i_*) of these processes is negligible, the equation collapses down to the following for the functions for *F(t)* and *R(t)*:

(9) $$F\left(t\right)={\left(1-e^{-\lambda t}\right)}^{N}⟹R\left(t\right)=1-{\left(1-e^{-\lambda t}\right)}^{N}$$

where N is the number of stages in a single ring. Each ring is described as a system of multiple elements. The *λ* is always much smaller than 1 assuming an early failure model: (t << 1/*λ*) [22]. We can therefore make the following approximation:

(10) $$e^{-\lambda t}\approx \;1-\lambda t.$$

So, based on the failure function in Equation (9) the reliability probability function is:

(11) R(t) ≈ 1−(λt)N ≈ e−(λt)N=e−(tθ)β.

The failure probability function is:

(12) $$F\left(t\right)=1-\;e^{-{\left(\lambda t\right)}^{N}}.$$

**Figure 11:**
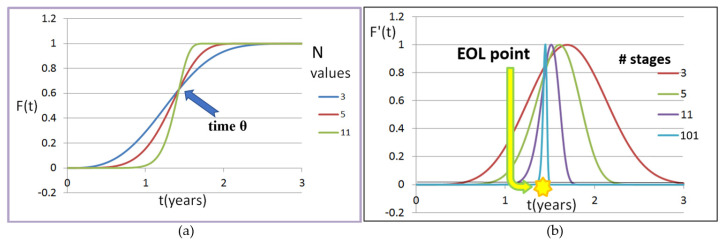
Plots of the failure distribution function defined by Equation 12 (**a**) pivoting around the θ point and the derivative of the failure distribution function defined by Equation 13 (**b**) revealing the end-of-life (EOL) point.

In Figure 11a, Equation (12) is plotted with N values of 3, 5, and 11. Note that the function flattens out at some point after θ. This is not an issue of concern since the system uses an extreme value approach as mentioned previously. The derivative of Equation (12) produces the failure distribution over time:

(13) $$F^{\prime }\left(t\right)=N\lambda {\left(\lambda t\right)}^{2N-1}*e^{-{\left(\lambda t\right)}^{N}}.$$

Figure 11b displays the result of Equation 13 for N values of 3, 5, 11, and 101. As the number of stages becomes larger, the gradient steepens. We have demonstrated both empirically and analytically that the shape of the failure distribution of a system of rings directly correlates to the number of stages programmed into the rings. Additionally, by averaging the degradation of many stages in a RO, very precise failure time or EOL is received.

## 3. The FLL Measurement Circuit

The reliability profiles generated with the MTOL method before this study used RO testing systems. Many other reliability testing methods use ROs as their degradation indicator [28,29,30,31]. In this study we present a highly accurate solution for chip performance monitoring over multiple frequencies. The motivation for changing the measurement circuit from standard ROs to the new FLL circuit is the RO’s lack the ability to control the ring frequency unless the number of ring stages is changed. The only way to generate high frequency is by implementing rings with few stages. Consequently, the precision of the TTF values received for these circuits will be poorer. This forces the designer to create a cumbersome amount of ring data to achieve a good average of TTF values, and thus a precise measurement. The disadvantages of using the RO solution are significantly increased in a health monitoring system. The TTF values must be calculated on the monitored microchip. To process the large data structures of TTF values, the microchip must perform heavy and resource costly computations. A health monitor is only a successful solution if it is resource efficient and transparent.

**Figure 12:**
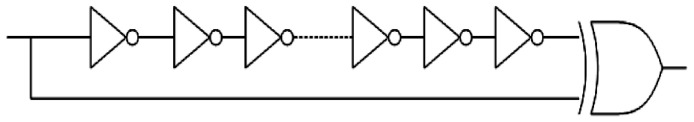
Diagram of a phase locked loop circuit.

Another configuration, the PLL circuit is used to monitor performance degradation in FPGAs [32,33]. The signal is forked at the beginning of the circuit. One route has an inverter chain and the other a free path. The measurement indicator is the shift in phase (see Figure 12). This allows testing of inverter chains to be any length desired. The downside of using the PLL circuit is that the phase drift is hardly discernible from the noise in the signal. We base this conclusion on results of PLL testing models performed on previous technologies. For this reason, the PLL circuit was not implemented on the technology tested. In contrast, frequency is a convenient parameter to measure microchip performance. Hence, we preferred to design a health monitoring system that uses frequency as its indicator.

In light of the above, a frequency monitored circuit where the number of stages and its level of frequency are controlled separately would be the optimal circuit for a performance degradation monitor circuit. This can take the pros of both the RO and PLL circuits. The implementation of this circuit design resolves the problem of TTF value dispersion in high frequencies. This solution facilitates the development of circuits with a large chain of inverters that can be stressed at high frequencies. As was demonstrated in the section above, such circuits produce very exact results.

**Figure 13:**
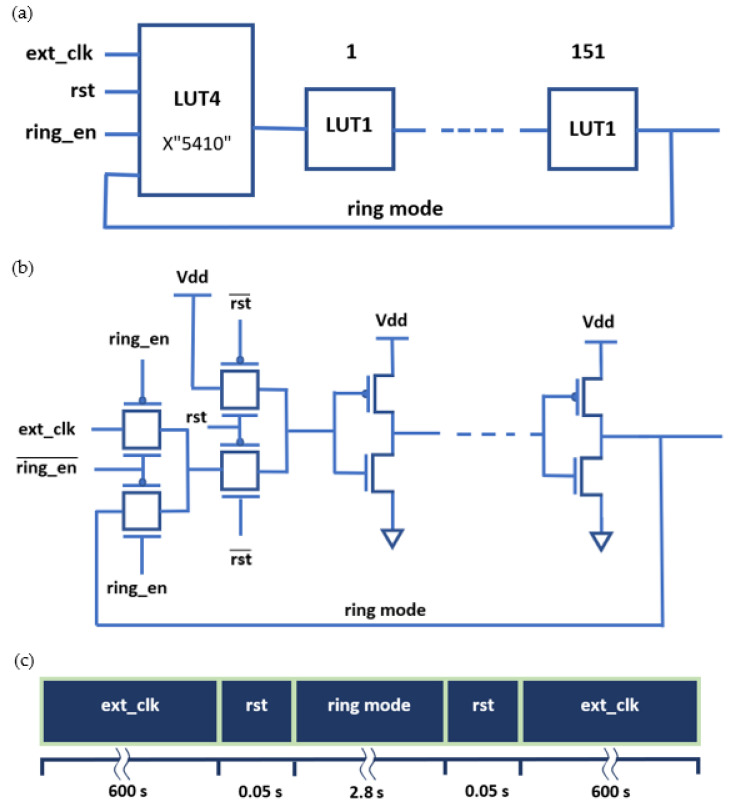
Diagram of the frequency locked loop (FLL) circuit: (**a**) Block diagram of lookup tables (LUTs). (**b**) The wire layout showing the connections of the circuit. (**c**) Timeline of the FLL operation cycle.

The FLL circuit offers stress frequency control without compromising on precision. The circuit operates in two modes: A stress mode and a measurement mode. For the stress mode, an external clock (ext_clk) delivers a predetermined frequency through the inverter stages that remain in an open chain. Since the inverters remain in this stress mode for relatively long period of time compared to the measurement time, the test can be considered in-situ or constantly stressed throughout the duration of the test. For the measuring stage, the circuit transitions into a ring oscillator (ring mode) for a short period. The frequency is sampled to observe the degradation trend. Between transitions, the circuit is reset (rst). Figure 13a details the logic layout programmed into the FPGA. A 4-input look-up table (LUT4) is connected to a chain of single input LUTs (LUT1). We chose to use 151 LUT1s to receive a good average. The transitions are initiated by a ring enable (ring_en) switch. Figure 13b shows a detailed wire diagram of the FLL circuit design. The design has two MUX layers that are connected to inverters. The logic of the two MUXs is using the Xilinx generate command in VHDL with the INIT in mode: X”5410”. The duration of the frequency stress is 10 min between measurements samples. This provides ratio of about 200 times more stress-on compared to stress-off. Figure 13c illustrates the time allocation of the different modes of the FLL.

**Figure 14:**
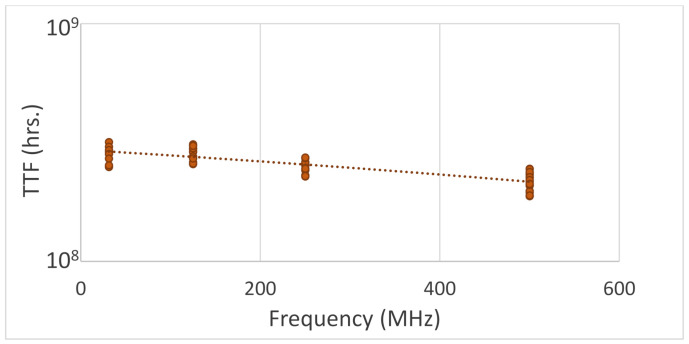
Three TTF to frequency plots averaged over the frequency. The decrease in TTF resembles the results received using ROs.

**Figure 15:**
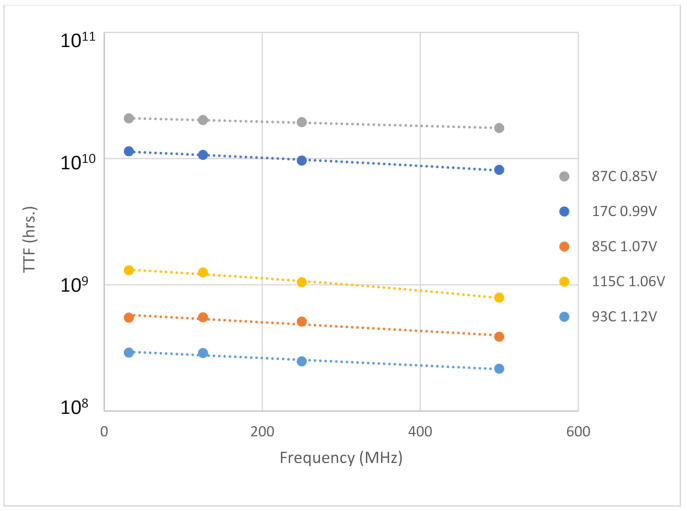
Five TTF to frequency plots averaged over the frequency. The decrease in TTF resembles the results received using ROs.

The FLL circuit was initiated on 16 nm FPGAs and MTOL tests were performed. The testing setup included 4 stress frequency modes: 31 MHz, 125 MHz, 250 MHz, and 500 MHz with 10 rings instantiated for each frequency mode. Figure 14 is an example plot of the TTF to frequency. The TTF values have a tight distribution which decreases with frequency increase. The results fit in line with the results performed using standard ROs. In Figure 15, the results of 5 tests using different stress conditions are displayed. The TTF values in each frequency node are averaged. The trend of TTF decrease with frequency is conserved for all the tests. The benefit of the FLL circuit is clearly seen by showing the contrast of the FLL results of Figure 14 to the RO results in Figure 3. In the Figure 3, the high frequency rings produce extremely dispersed TTF values. In Figure 14, the results throughout all the frequencies retain tight distributions.

## 4. The Microchip Health Monitor

**Figure 16:**
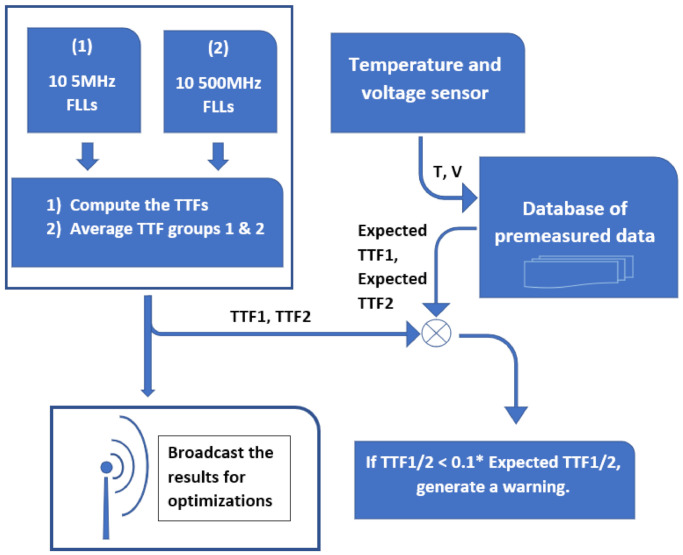
An operation flow of the MCM system.

The MHM system is formulated using the concepts detailed in the previous sections. According to reliability trends in the latest technologies, stress due to low temperatures is no longer a factor since there is an acute reduction in HCI. Since in high temperatures there is only one dominant mechanism, BTI, separation of failure mechanisms is not necessary. The MHM system has the lab data tested using the MTOL testing method stored in a database. Figure 16 displays the flow of the monitor. The following parameters are measured every 10 min: The ring frequency of the 10 FLL circuits with an external clock of 5 MHz, the ring frequency of the 10 FLL circuits with an external clock of 500 MHz, internal voltage, and temperature. The TTF values are calculated using Equations (3) and (4) listed on page 5 and averaged. The averaged TTF results called TTF1 and TTF2 are compared to the TTF values for the two frequencies stored in the database. The precision of the FLL circuits allow minimal sensitivity for the measured TTF values. If there is a decrease in TTF of a full order of magnitude, the monitored micro-chip with generate a warning flag. Given that the MCM system is implemented in a large scale of devices, the results will be broadcasted to a central hub to optimize the formula of the database data in the future. Having such a system in advanced infrastructures can allow cautioning of harmful chip degradation before damage is caused.

## 5. Conclusions

We have proposed a design for precise microchip degradation monitoring on up-to-date devices. The concept uses real data from packaged devices to develop an early warning system that is space efficient and transparent to the user. We show that current transistor technologies are designing out HCI and EM failure mechanisms making BTI the dominant cause for degradation. The novel FLL measurement circuit assists the design to work with minimal data and maximum accuracy. The design figures in a module for optimizing the control data from the data collected from the monitor. The MHM system can be implemented on virtually any FPGA or ASIC chip.

## Data Availability

The data presented in this study are available on request from the corresponding author.

## References

[1] Bernstein J.B., Bensoussan A., Bender E. (2020). Reliability Prediction of FinFET FPGAs by MTOL. Microelectron. Reliab..

[2] Bernstein J.B., Bensoussan A., Bender E. (2017). Reliability prediction with MTOL. Microelectron. Reliab..

[3] Hu C., Tam S.C., Hsu F.-C., Ko P.-K., Chan T.-Y., Terrill K.W. (1985). Hot-carrier-induced MOSFET degradation—Model, monitor, and improvement. IEEE Trans. Electron Dev..

[4] Acovic A., Rosa G.L., Sun Y.-C. (1996). A review of hot-carrier degradation mechanisms in MOSFETs. Microelectron. Reliab..

[5] Lee Y., Mielke N., Agostinelli M., Gupta S., Lu R., McMahon W. Prediction of Logic Product Failure due to Thin-Gate Oxide Breakdown. Proceedings of the 2006 IEEE International Reliability Physics Symposium.

[6] Deal B.E. (1974). The current understanding of charges in the thermally oxidized silicon structure. J. Electrochem. Soc..

[7] Young D., Christou A. (1994). Failure mechanism models for electromigration. IEEE Trans. Reliab..

[8] Hau-Riege C.S., Thompson C.V. (2001). Electromigration in Cu interconnects with very different grain structures. Appl. Phys. Lett..

[9] Bernstein J.B., Gabbay M., Delly O. (2014). Reliability matrix solution to multiple mechanism prediction. Microelectron. Reliab..

[10] Bernstein J.B. (2014). Reliability Prediction from Burn-In Data Fit to Reliability Models.

[11] Rashkeev S.N., Fleetwood D.M., Schrimpf R.D., Pantelides S.T. (2001). Proton-induced defect generation at the Si-SiO/sub 2/ interface. IEEE Trans. Nucl. Sci..

[12] Rangan S., Mielke N., Yeh E.C.C. Universal recovery behavior of negative bias temperature instability [PMOSFETs]. Proceedings of the IEEE International Electron Devices Meeting 2003.

[13] Varghese D., Mahapatra S., Alam M.A. (2005). Hole energy dependent interface trap generation in MOSFET Si/SiO/sub 2/ interface. IEEE Electron Device Lett..

[14] Huard V., Denais M., Parthasarathy C. (2006). NBTI Degradation: From Physical Mechanisms to Modeling. Microelectr. Reliab..

[15] Qu Y., Lin X., Li J., Cheng R., Zheng Z., Lu J., Chen B., Zhao Y. Ultra Fast (<1 ns) Electrical Characterization of Self-Heating Effect and Its Impact on Hot Carrier Injection in 14 nm FinFETs. Proceedings of the 2017 IEEE International Electron Devices Meeting (IEDM).

[16] Zhang G., Gu Y., Li J., Tao H. (2015). An Improved Model of Self-Heating Effects for Ultrathin Body SOI nMOSFETs Based on Phonon Scattering Analysis. IEEE Electron Device Lett..

[17] Makovejev S., Olsen S., Raskin J.-P. (2011). RF Extraction of Self-Heating Effects in FinFETs. IEEE Trans. Electron Dev..

[18] Djemouai A., Sawan M.A., Slamani M. (2001). New Frequency-Locked Loop Based on CMOS Frequency-to-Voltage Converter: Design and Implementation. IEEE Trans. Circuits Syst. II Analog Digit. Signal Process..

[19] Alam M.A., Kufluoglu H., Varghese D., Mahapatra S. (2007). A comprehensive model for PMOS NBTI degradation: Recent progress. Microelectron. Reliab..

[20] Kufluoglu H., Alam M. (2004). A Computational Model of NBTI and Hot Carrier Injection Time-Exponents for MOSFET Reliability. J. Comput. Electron..

[21] Jeppson K.O., Svensson C.M. (1977). Negative bias stress of MOS devices at high electric fields and degradation of MNOS devices. J. Appl. Phys..

[22] Weibull W. (1951). A Statistical Distribution Function of Wide Applicability. J. Appl. Mech..

[23] Gall M., Capasso C., Jawarani D., Hernandez R., Kawasaki H. (2001). Statistical Analysis of Early Failures in Electromigration. J. Appl. Phys..

[24] Klutke G., Kiessler P.C., Wortman M.A. (2003). A Critical Look at the Bathtub Curve. IEEE Trans. Reliab..

[25] Drenick R.F. (1960). The Failure Law of Complex Equipment. J. Soc. Ind. Appl. Math..

[26] Myers R.H., Wong K.L., Gordy H.M. (1964). Reliability Engineering for Electronic Systems.

[27] Bisschop J. (2007). Reliability Methods and Standards. Microelectron. Reliab..

[28] Hsieh M.H., Huang Y.C., Yew T.Y., Wang W., Lee Y.H. The Impact and Implication of BTI/HCI Decoupling on Ring Oscillator. Proceedings of the 2015 IEEE International Reliability Physics Symposium.

[29] Huang Y., Yew T., Wang W., Lee Y., Shih J.R., Wu K. Re-investigating the Adequacy of Projecting Ring Oscillator Frequency Shift from Device Level Degradation. Proceedings of the 2014 IEEE International Reliability Physics Symposium.

[30] Huang Y., Yew T., Wang W., Lee Y., Shih J.R., Wu K. Re-investigation of Frequency Dependence of PBTI/TDDB and Its Impact on Fast Switching Logic Circuits. Proceedings of the 2013 IEEE International Reliability Physics Symposium (IRPS).

[31] Wang X., Jain P., Jiao D., Kim C.H. Impact of Interconnect Length on BTI and HCI Induced Frequency Degradation. Proceedings of the 2012 IEEE International Reliability Physics Symposium (IRPS).

[32] Kim K.K., Wang W., Choi K. (2010). On-Chip Aging Sensor Circuits for Reliable Nanometer MOSFET Digital Circuits. IEEE Trans. Circuits Syst. II Express Briefs.

[33] Stott E., Guan Z., Levine J., Wong J., Cheung P. (2013). Variation and Reliability in FPGAs. IEEE Des. Test.

